# 3D-MRCP for evaluation of intra- and extrahepatic bile ducts: comparison of different acquisition and reconstruction planes

**DOI:** 10.1186/1471-2342-14-16

**Published:** 2014-05-19

**Authors:** Kristina Imeen Ringe, Dagmar Hartung, Christian von Falck, Frank Wacker, Hans-Jürgen Raatschen

**Affiliations:** 1 Department of Diagnostic and Interventional Radiology, Hannover Medical School, Carl-Neuberg Str. 1, Hannover 30625, Germany

**Keywords:** MRCP, MRC, Magnetic resonance cholangiopancreatography, Bile ducts, Common hepatic duct

## Abstract

**Background:**

Magnetic resonance cholangiopancreatography (MRCP) is an established technique for the evaluation of intra- and extrahepatic bile ducts in patients with known or suspected hepatobiliary disease. However, the ideal acquisition and reconstruction plane for optimal bile duct evaluation with 3D technique has not been evaluated. The purpose of our study was to compare different acquisition and reconstruction planes of 3D-MRCP for bile duct assessment.

**Methods:**

34 patients (17f/17 m, mean age 41y) referred for MRCP were included in this prospective IRB-approved study. Respiratory-triggered 3D-T2w-MRCP sequences were acquired in coronal and axial plane. Coronal and axial MIP were reconstructed based on each dataset (resulting in two coronal and two axial MIP, respectively). Three readers in two sessions independently assessed the MIP, regarding visualization of bile ducts and image quality. Results were compared (Wilcoxon test). Intra- and interobserver variability were calculated (kappa-statistic).

**Results:**

In case of coronal data acquisition, visualization of bile duct segments was significantly better on coronal reconstructed MIP images as compared to axial reconstructed MIP (p < 0.05). Regarding visualization, coronal MIP of the coronal acquisition were equal to coronal MIP of the axial acquisition (p > 0.05). Image quality of coronal and axial datasets did not differ significantly. Intra- and interobserver agreement regarding bile duct visualization were moderate to excellent (κ-range 0.55-1.00 and 0.42-0.85, respectively).

**Conclusions:**

The results of our study suggest that for visualization and evaluation of intra- and extrahepatic bile duct segments reconstructed images in coronal orientation are preferable. The orientation of the primary dataset (coronal or axial) is negligible.

## Background

Magnetic resonance cholangiopancreatography (MRCP) is an established technique for the evaluation of intra- and extrahepatic bile ducts in patients with known or suspected hepatobiliary disease [1]. It is considered a reliable non-invasive alternative to diagnostic endoscopic retrograde cholangiopancreatography (ERCP) [2,3]. Since the first description by Wallner and colleagues in 1991 [4], different acquisition techniques have evolved.

Most current MRCP techniques are based on heavily T2-weighted fast spin echo (FSE) pulse sequences, which yield a luminal image of the bile ducts that is based on the inherent signal of slow-flowing or stationary bile. Both, single shot projections and multislice techniques are available [5], with the latter being distinguished into 2D [6] and 3D techniques [7]. Single shot projections are preferred in individuals who are unable to hold their breath, such as severely sick patients or small children [7]. 3D imaging techniques provide better image quality compared to 2D sequences [1,8,9], even though the combination of different MRCP sequences has proven to be valuable in the assessment of bile duct anatomy and pathology [10].

3D FSE sequences are usually acquired with the slab in coronal orientation. Maximum intensity projections (MIP) can then be obtained in any plane [7]. Previous studies have addressed the matter of optimal slice thickness for data acquisition [11] and different techniques regarding respiratory triggering [12]. However, to the best of our knowledge, the ideal acquisition and reconstruction plane, in practical terms meaning best suitable for optimal bile duct visualization with 3D techniques, has not been evaluated. The purpose of this study was to compare different acquisition and reconstruction planes of T2-weighted 3D-MRCP acquisitions for assessment of the intra- and extrahepatic bile ducts.

## Methods

### Patients

This HIPAA-compliant study was approved by the institutional review board of Hannover Medical School. Written informed consent from each patient was obtained. 34 patients (17 female, 17 male, mean age 41.5 years, range 18-79 years) who were referred for liver MRI and dedicated MRCP were included in this prospective study. Inclusion criteria were as follows: completion of the entire MR examination; patient age equal or greater than 18 years. Indications for performing MRI were as follows: primary sclerosing cholangitis (n = 16), status post liver transplantation (n = 7), tumor (n = 6), suspicion of Caroli’s disease (n = 2), stone disease (n = 2) and recurrent pancreatitis (n = 1).

### MR imaging technique

MR examinations were performed on a 1.5 T system (Magnetom Avanto, Siemens, Erlangen, Germany) using dedicated multichannel surface coils covering the abdomen. Prior to image acquisition, patients received 200 mL of a negative oral contrast agent (Lumirem®; Guerbet, Sulzbach, Germany) for suppression of gastroenteric fluid signal, as well as 20 mg butylscopolamine (Buscopan®; Boehringer Ingelheim, Ingelheim, Germany; administered as a bolus over approximately 20 s) i.v. for spasmolysis. All patients underwent a clinical routine imaging protocol of the liver, including a respiratory-triggered 3D MR cholangiography in the coronal (dataset A) as well as in the axial (dataset B) plane. MRCP-sequences were acquired prior to intravenous contrast injection. The specific MRCP sequences had sequential k-space filling with partial Fourier filling allowed, resulting in acquisition of central k-space lines approximately 3 minutes after the start of the sequence. MRCP sequence parameters are provided in detail in Table 1.

**Table 1:** Imaging parameters of the respiratory-triggered fat-saturated 3D T2-weighted MR cholangiographic sequence

**Parameter**	**1.5 T Magnetom Avanto**
Plane	Coronal, Axial
Respiratory triggering	Navigator based
Repetition time	Breathing cycle
Echo time (msec)	700-800
Refocusing pulse	140°
In-plane spatial resolution (mm)	1 × 1
Slab thickness (mm)	60-80
Partitions per slab	60-80
Type of k-space filling	Sequential
Partial Fourier factor	Allowed
Estimated total acquisition time (min)	Approximately 5

### Image evaluation

Three readers (D.H., C.F., H.J.R.) independently performed image evaluation in terms of visibility of different bile duct segments up to the 3^rd^ order and assessment of technical quality. All readers were board certified radiologists with at least eight years of experience in abdominal MR imaging. Readers were blinded to each patient’s history and other imaging findings.

A single coronal and axial maximum intensity projection (MIP) covering the central, left, right and peripheral bile ducts was generated from each acquired MRCP dataset, resulting in two coronal and two axial MIP datasets, respectively. Care was taken to exclude the renal pelvis, ureter of both kidneys and spinal canal to allow for blinded reading. Each reader performed two reading sessions separated by an interval of two weeks, evaluating the reconstructed MIP in the following way: 1, Coronal reconstructed MIP of the coronal acquisition vs. coronal reconstructed MIP of the axial acquisition; 2, Axial reconstructed MIP of the coronal acquisition vs. axial reconstructed MIP of the axial acquisition. After each reading session the readers were asked to choose the preferred image dataset of any given comparison. The readers had no knowledge of initial MRCP dataset orientation.

Depiction of bile duct segments was assessed by using the following four-point scale proposed by Papanikolaou and colleagues [13]: 1, segment not seen; 2, segment faintly seen; 3, segment well seen but portion of the duct or the confluence not seen; and 4, excellent depiction including the proximal and distal portions. This scale was applied to the following sections (segments) of the biliary tract: the common bile duct (CBD), the right anterior bile duct, the right posterior bile duct, the left hepatic duct, and third-order biliary branches. Overall technical image quality was assessed using a four-point scale proposed by Lim and colleagues [14]: 1, poor quality with severe artifacts; 2, satisfactory quality with few artifacts; 3, good quality with minimal artifacts; and 4, excellent quality without artifacts.

In addition, a total score was defined as the sum of the visibility and quality scores for each dataset to determine which of the two acquired MRCP datasets (coronal or axial) yielded the most diagnostic information.

### Statistical analysis

Statistical analysis was performed using GraphPad Prism software (version 6; GraphPad Software, Inc., La Jolla, CA).

Results regarding bile duct visualization and overall technical image quality were compared with a two-sided Wilcoxon signed-rank test (with a p-value <0.05 deemed significant) in the following way: 1, Coronal reconstructed MIP of the coronal acquisition vs. coronal reconstructed MIP of the axial acquisition; 2, Axial reconstructed MIP of the coronal acquisition vs. axial reconstructed MIP of the axial acquisition; 3, Coronal vs. axial reconstructed MIP of the coronal acquired dataset; 4. Coronal vs. axial reconstructed MIP of the axial acquired dataset. Intra- and interobserver agreement was assessed by means of a kappa-statistic and classified as follows: a Κ value of less than 0.20 indicated poor agreement; Κ values of 0.21-0.40, fair agreement; Κ values of 0.41-0.60, moderate agreement; Κ values of 0.61-0.80, good agreement; and Κ values of 0.80-1.00, excellent agreement [14].

## Results

### Bile duct visualization

In case of coronal data acquisition (dataset A), visualization of bile duct segments was significantly better on coronal reconstructed MIP as compared to axial reconstructed MIP (p < 0.05). This was true for visualization of the CBD, right anterior hepatic duct, left hepatic duct and third order biliary branches. In case of axial data acquisition (dataset B), a significant better visualization of the CBD and left hepatic duct on coronal reconstructed MIP as compared to axial reconstructed MIP was observed only by one reader.

**Figure 1:**
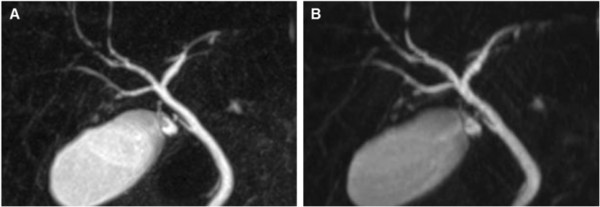
**Comparison of coronal MIP reconstructions of coronal and axial acquired datasets.** 35-year old male patient with diagnosis of primary sclerosing cholangitis (PSC). Coronal MIP of a coronal **(A)** and axial **(B)** acquired MRCP dataset. Bile duct visualization up to the 3^rd^ order is equal on both datasets (see also Table 2), even though the image impression is more blurred on the MIP derived from the axial acquired dataset **(B)**.

**Figure 2:**
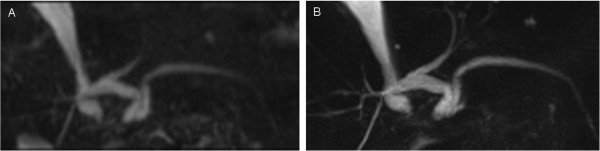
**Comparison of axial MIP reconstructions of coronal and axial acquired datasets.** 79-year old male patient status post radiofrequency ablation of a colorectal liver metastasis. Axial MIP of a coronal **(A)** and axial **(B)** acquired MRCP dataset. Bile duct visualization of 1^st^ and 2^nd^ order branches is equal on both datasets, whereas 3^rd^ order branches are depicted significantly better on the axial dataset.

**Figure 3:**
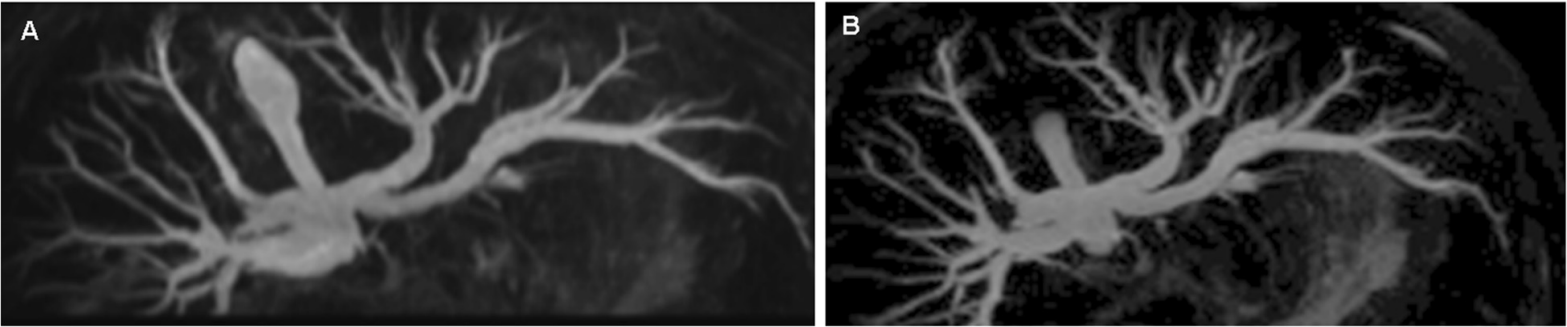
**Comparison of axial MIP reconstructions of coronal and axial acquired datasets.** 18-year old male patient with jaundice and suspicion of cholelithiasis. Axial MIP of a coronal **(A)** and axial **(B)** acquired MRCP dataset. Bile duct visualization up to the 3^rd^ order is equal on both datasets, even though the image impression is more blurred on the MIP derived from the coronal acquired dataset **(A)**.

Regarding bile duct visualization, coronal MIP of the coronal acquisition (dataset A) were equal to coronal MIP of the axial acquisition (dataset B) (p > 0.05) (Figure 1; Table 2). Axial MIP of the axial acquisition (dataset B) were significantly better than axial MIP of the coronal acquisition (dataset A) for visualization of third order biliary branches whereas lower order branches did not show a difference (Figures 2 and 3).

**Table 2:** P-values for each reader and biliary segment: comparison of coronal reconstructed MIP of coronal and axial acquired datasets regarding bile duct visualization

**Reader No. (Session No.)**	**CBD**	**Right posterior bile duct**	**Right anterior bile duct**	**Left hepatic duct**	**2**^**nd **^**and 3**^**rd **^**order branches**
1 (1)	0.424	0.923	0.499	0.685	0.305
1 (2)	0.766	0.309	0.236	0.783	0.790
2 (1)	1.000	1.000	0.536	0.358	0.145
2 (2)	1.000	0.609	0.400	0.891	0.393
3 (1)	0.174	0.943	0.305	0.266	0.627
3 (2)	0.345	0.898	0.143	0.627	0.608

P values were calculated with the two-sided Wilcoxon Test to compare depiction scores of coronal axial acquired datasets.

Intraobserver agreement regarding bile duct visualization was good to excellent (weighted Κ-range 0.63-1.0). Interobserver agreement was moderate to good, regarding bile duct visualization in both datasets (coronary acquisition: weighted Κ range 0.51-0.75; axial acquisition: weighted Κ range 0.42-0.67).

### Technical image quality

Regarding overall technical image quality (including axial and coronal reconstructed MIP of a given dataset), there was no significant difference between the coronal and axial acquired dataset (p > 0.05). At detailed dataset analysis however, in case of coronal data acquisition (dataset A) technical image quality of the coronal MIP was significantly better as compared to the axial reconstructed MIP (p < 0.05). In case of axial data acquisition (dataset B), there was no significant difference regarding technical image quality of the reconstructed MIP (p > 0.05).

Intraobserver agreement regarding technical image quality was moderate to excellent (weighted Κ range 0.55-0.96); interobserver agreement was moderate (weighted Κ range 0.42-0.59).

### Choice of preferred image dataset

When reading coronal reconstructed MIP, the readers preferred coronal acquisitions over axial acquisitions in 66% of the readings. Regarding axial MIP reconstruction, axial acquisitions were preferred over coronal acquisitions in 80% of the readings.

Intraobserver agreement regarding choice of the preferred image dataset was excellent (weighted Κ range 0.94-1.00); interobserver agreement was moderate to excellent (weighted Κ range 0.57-0.85).

## Discussion

To the best of our knowledge the ideal acquisition and reconstruction plane for optimal bile duct evaluation with 3D techniques has not yet been evaluated. For single shot FSE techniques it has been suggested that straight coronal and initial left posterior oblique images clearly depict the common hepatic duct and the left hepatic duct, whereas the CBD and right hepatic ducts are seen better on a left posterior images obtained at a steeper angle [15]. Especially in children with segmental liver transplants sagittal oblique planes are preferred due to the more anteroposterior orientation of the neo-porta hepatis [16]. In a first approach towards projection cholangiography by means of MRI in 1991, Wallner and colleagues used a heavily T2-weighted gradient echo sequence for assessment of bile duct dilatation [4]. They concluded that imaging in the coronal plane provided a good view of the biliary system, whereas no additional information was found by imaging in the sagittal plane.

In this study we compared different acquisition and reconstruction planes of T2-weighted 3D-MRCP acquisitions for assessment of the intra- and extrahepatic bile ducts. In contrast to single shot techniques, 3D MRCP has the advantage to facilitate secondary reconstructions. Coronal reconstructions were preferred, regardless of the initial acquisition plane. These findings were supported by good intra- and interobserver agreement. One of the reasons for coronal image preference might be the fact that these images are similar to image impressions of ERCP and conventional cholangiograms.

There are other studies that evaluated secondary reconstruction techniques for MRCP. Schaible and colleagues evaluated selective MIP reconstructions of respiratory-triggered 3D MRCP versus standard MIP reconstructions and single-shot MRCP [17]. Single-shot and standard MIP reconstructions of 3D MRCP were comparable in terms of anatomical bile duct visualization, whereas selective MIP postprocessing proved useful for detection of pathological alterations. In a retrospective study, Morita and colleagues compared volume rendering (VR) and MIP of 3D TSE MRCP sequences to define biliary anatomy mostly in patients without major biliary tract anomaly [18]. Definition of biliary anatomy was found to be more accurate using VR reformation than MIP. However, the assessment of VR images was not the purpose of the present study. One disadvantage of VR reconstructions is that the detection degree of each structure depends on the setting of display parameters, particularly on the lower threshold of the opacity curve. Therefore VR images need to be evaluated interactively [18].

Our study had some limitations. We did not perform quantitative SNR measurements of the MRCP datasets as the focus of our study was to qualitatively evaluate visualization of intra- and extrahepatic bile ducts using multiple readers. This seemed closer to the clinical reality than SNR values that are difficult to measure for small bile ducts. We did not evaluate the added value of acquisition or reconstruction planes. In 1999, Boraschi and colleagues compared axial and coronal 2D FSE sequences with 3D MIP projection images in patients with suspected hepatobiliary disease [19]. A higher global accuracy for axial and coronal FSE T2-weighted sequences was found regarding the diagnosis of the level and probable cause of biliary obstruction in depiction of small intraductal pathology such as calculi or neoplastic lesions.

We have limited our analysis to reconstructed rather thin-slice source images as the purpose of this specific was to directly compare acquisition and reconstruction planes for MIP assessment. A well-known limitation of MIP is that small filling defects may be obscured due to partial volume effects [20]. Further, overestimation of ductal narrowing and pseudostricture may result from the nature of MIP reconstruction [21]. Therefore it is important, that MIP reconstructions should not be appraised separately, but always in combination with the original acquired dataset and in combination with other morphological sequences.

## Conclusions

In conclusion, we compared different acquisition and reconstruction planes of T2-weighted 3D-MRCP acquisitions for assessment of the intra- and extrahepatic bile ducts in patients with different hepatobiliary pathologies. The results of our study suggest that for visualization and evaluation of the bile ducts coronal reconstructions are preferred. In this context, the orientation of the primary dataset (coronal or axial) is negligible.

## Competing interests

The authors declare that they have no competing interests.

## Authors’ contributions

KIR and DH conceived and designed the experiments, KIR, DH and CF performed the experiments and acquisition of data. KIR, DH, CF, FW and HJR analyzed and interpreted the data. FW and HJR contributed materials and analysis tools. All authors participated in drafting and revising the manuscript. All authors read and approved the final manuscript.

## Pre-publication history

The pre-publication history for this paper can be accessed here:

http://www.biomedcentral.com/1471-2342/14/16/prepub
